# CO_2_ Plasticization Resistance Membrane for Natural Gas Sweetening Process: Defining Optimum Operating Conditions for Stable Operation

**DOI:** 10.3390/polym14214537

**Published:** 2022-10-26

**Authors:** Farahdila Kadirkhan, Goh Pei Sean, Ahmad Fauzi Ismail, Wan Nurul Ffazida Wan Mustapa, Mohd Hanif Mohamad Halim, Soh Wei Kian, Yeo Siew Yean

**Affiliations:** 1 Advanced Membrane Technology Research Centre (AMTEC), Faculty of Chemical and Energy Engineering, Universiti Teknologi Malaysia, Johor Bahru 81310, Malaysia; 2 Carbon Capture Utilisation & Storage (CCUS) R&D Programme, Group Research & Technology (GR&T), Project Delivery & Technology (PD&T), PETRONAS Research Sdn Bhd (PRSB), Block E, Lot 3288 & 3289, Off Jalan Ayer Itam, Kawasan Institusi Bangi, Kajang 43000, Malaysia

**Keywords:** CO_2_ removal, field performance evaluation, mixed gas, operating pressure and temperature, CO_2_ percentage variation

## Abstract

Membranes with a stable performance during the natural gas sweetening process application are highly demanded. This subject has been immensely explored due to several challenges faced by conventionally used polymeric membranes, especially the high tendency of plasticization and physical aging. In this study, polysulfone (PSf) hollow-fiber membrane was formulated and tested for its application in natural gas sweetening based on several compositions of CO_2_/CH_4_ mixed gas. The effects of operating conditions such as pressure, temperature and CO_2_ feed composition on separation performance were analyzed. The findings showed that the formulated membrane exhibited decreasing CO_2_ permeation trend with the increase in pressure. Conversely, the increase in operating temperature boosted the CO_2_ permeation. High productivity can be attained at higher operating temperatures with a reduction in product purity. Interestingly, since PSf has higher plasticization pressure, it was not affected by the change in CO_2_ percentage up to 70% CO_2_. The experimental study showed that the membrane material formulated in this study can be potentially evaluated at the field stage. Longer testing duration is needed with the real feed gas, appropriate pre-treatment based on the material limitations, and optimum operating conditions at the site to further confirm the membrane’s long-term lifetime, resistance, and stability.

## 1. Introduction

Gas separation is an attractive technology for a wide range of chemical processes. Gas separation membranes have been predicted to have a compound annual growth rate (CAGR) of 5.87% by 2027 [1]. Asia Pacific represents the largest and fastest growing market, with increasing consumption predicted from countries such as Japan, India, and China [1]. The global gas separation membrane market was valued at USD 957.47 million in 2021 [1], owing to the efforts of many governments to decrease greenhouse gas emissions in order to mitigate issues related to global warming [2]. Using a gas separation membrane, the permeate, which consists mainly of CO_2_ gas, can be injected into depleted reservoirs to reduce its emission or improve oil and shale oil recovery. CO_2_ is miscible with oil and can induce oil swelling hence reducing its viscosity. Many studies have shown that CO_2_ can be used for enhanced oil and gas recovery and geological storage through lab experiments, field tests, molecular and reservoir simulation [3,4].

The membrane materials used for commercial application are mainly cellulose acetate, PSf, and polyimide. However, the classical polymeric membrane often suffers from plasticization, which leads to lower performance [5]. The use of polyimide polymer for the separation of CO_2_ has attracted much commercial interest. However, the particular concern is to prevent and suppress the plasticization of this polymer [6]. Plasticization occurs mainly due to the presence of plasticizers in medium to high concentrations [7]. Among the plasticizers for natural gas application are the non-polar gas CO_2_ and heavy hydrocarbon.

High operating pressure beyond the material plasticization pressure [8,9,10,11] and high operating temperature [6] can further worsen membrane plasticization. The effect of temperature on plasticization can significantly affect diffusivity. This is explained by an Arrhenius relationship with temperature and a relationship with penetrant concentration which follows an exponential trend [6]. Under mixed gas conditions at alleviated pressure, the CO_2_/CH_4_ selectivity of cellulose acetate polymer often drops to less than 15 due to multiple phenomena such as aging, competitive sorption, and plasticization. This leads to marginally acceptable project economics due to the unnecessary loss of valuable methane gas [6,8].

Plasticization makes the polymer swell, and this slight solvation can permanently damage the polymer structure. Inter-chain spacing becomes larger hence increasing the gas and vapor diffusion coefficients [6]. Plasticization has been correlated to polymer chain softening, which results in the loss of separation ability [9], deteriorated material thermal and mechanical properties, and accelerated polymer aging [10]. Membrane aging leads to densification and free volume reduction. Thus, the gas permeability is reduced while the selectivity is enhanced or maintained [11]. During densification, the free volume is believed to undergo “lattice contraction,” which reduces gas flow [12,13].

Membranologist are constantly looking for solutions to achieve an optimum gas permselective membrane while maintaining a good balance between selectivity and permeation [14]. Currently, among the strategies highlighted in the literature to tackle plasticization issues, using the long chain polymer with nonordered structure, using highly crystalline polymer and polymer with high molecular weight, performing thermal or chemical cross-linking to the polymer, improving hydrogen bonding of the polymer by incorporating CO_2_-philic groups into the polymer, enhancing polymer stiffness by including per substituted rings, incorporating bulky side groups to lessen the rotational ability, and eliminating any flexible linkages, are included [7,15,16]. A polymer modification method such as grafting has also been used to improve the polymer resistance against plasticization [17] and enhance membrane performance [18]. Grafting can be performed by using sulfuric compounds, carboxyl, hydroxyl, and amines [19], as well as through the introduction or exchange of small functional groups onto the polymer’s rotating unit [17].

Alaslai fabricated polyimide membranes functionalized with hydroxyl groups [8]. From the high-pressure test up to 40 atm using a binary feed gas of 50:50 CO_2_/CH_4_, they found that the membrane exhibited lower CO_2_ permeability compared to single gas testing due to competitive sorption, while CH_4_ permeability remained unchanged. The selectivity was reduced from 70 to 41 (40% reduction) when the 4 atm feed pressure increased to 40 atm for 6 FDA-mPDA (m-phenylene diamine). The other type of hydroxyl-functionalized polyimides, 6 FDA-DAR (diamino resorcinol) and 6 FDA-DAP (diaminophenol) also showed similar behavior with a reduction of 30% for the selectivity when the pressure was increased. The competition effect from other gases such as the one shown in this study by Alaslai, can only be observed when multi-component testing is performed [20].

Visser performed some experimental studies using various CO_2_ and CH_4_ mixtures for four types of asymmetric membranes. It was determined that plasticizing Matrimid and cellulose acetate membranes result in a significant loss of selectivity [21]. Falbo found out that the separation factor of polyimide membrane tested with gas mixture was less by 20% compared to single gas in the temperature range of 25 °C to 75 °C under pressure up to 600 kPa. When the gas was saturated with water, competitive sorption reduced the permeation of all gases [22].

Ricci studied the multi-component gas separation performance of cellulose acetates membrane. They found that the competitive sorption enhanced the solubility selectivity while overall perm-selectivity was reduced due to a reduction in diffusivity selectivity that was largely impacted. From 0 to 5 MPa pressure, the CO_2_/CH_4_ solubility selectivity increased from 16 and was maintained at 17 for equimolar CO_2_ and CH_4_, while CO_2_/CH_4_ diffusivity selectivity was reduced from 2 to 1.5. This resulted in the overall CO_2_/CH_4_ perm-selectivity being reduced from 30.5 to 24.5. CO_2_-induced swelling caused a faster diffusion of CH_4_ in the mixed gas case. CTA (Cellulose Tri-Acetate) was found to be less susceptible to the detrimental effects of CO_2_-induced swelling, which caused a reduction of the diffusivity-selectivity since its solubility-selectivity is more dominant. CTA perm-selectivity also displayed a weaker dependence on the CO_2_ content in the mixture [23].

Miandoab performed a simulation of multi-component gas, including water, in humidified versus dry feed conditions. The membrane model shows that a simplistic model that does not account for such complex effects can deviate more than 20% from a more rigorous approach. The impact of competitive sorption and plasticization by CO_2_ and H_2_O-induced free volume blocking was significantly greater, leading to fugacity-dependent permeabilities for CO_2_ and CH_4_ that were below those of the pure gas components. Relative to the simplified models, the new model predicts differences up to 2% and 18% in CH_4_ recovery at low feed flow rates, and the difference in CO_2_ removal can be as significant as 50% [24].

Genduso found that the permeability selectivity of the mixture strongly diverges from the pure-gas trend. At about 18 atm partial fugacity, almost 35% of the ideal permeability selectivity was lost, and it was assumed that competitive sorption was the cause for this loss of permeability selectivity. The data of mixed-gas solubilities at 50 mol% equilibrium concentration showed that competitive sorption strongly affects CH_4_ mixed-gas solubility, that is, when partial pressures increase, the CH_4_ mixed-gas solubility coefficients diverge from the pure-gas values. Because the effect of competitive sorption on the solubility coefficient of CO_2_ is limited, they found that at 2 atm CO_2_ partial pressure, the CO_2_/CH_4_ solubility selectivity increased from ~5 in the pure-gas state to ~10 in the mixture [25].

Operating conditions such as pressure, temperature, and flow rate can influence the resistance of the membrane during its application which will, in turn, impact the working lifetime of the membrane. The product purity and productivity are determined by various factors such as membrane properties, which are also helped by selecting the optimum operating conditions [26]. The accurate choice of the best-operating conditions enables the membrane to function continuously with good performance for years with minimal hydrocarbon and permeance loss over time.

Most previous works reported were based on CO_2_ and CH_4_ separation using low pressure with single gas and simple binary gas. Multi-component gas mixture permeation properties were not widely studied due to the difficulty and risk of handling the operating parameters at a higher level and the need to analyze more than two components simultaneously [14]. Single gas testing has overlooked the effects of competitive sorption, plasticization, compaction, pore blocking, and aging, which upset the membrane performance when tested in the field under harsh conditions. Furthermore, raw natural gas usually contains heavy hydrocarbon [27], and the CO_2_ contaminant concentration may vary from field to field in the range of 15% to 70%. For less than 15% CO_2_, the absorption technology is usually applied.

The objective of this research was to study the performance of a formulated glassy PSf membrane when subjected to various operating conditions. The aim was to identify the optimum setting that will be used for its field application with stable operation. Experiments using pure and mixtures of five gases typically found in natural gas with various compositions were carried out. Effects of operating temperature (25 to 45 °C), pressure (20 to 50 barg), and CO_2_ composition (15%, 20%, 40% and 70%) variation were investigated. The effect of operating conditions on the rubbery membrane can be compared and is referred to the study conducted by Sadrzadeh [14]. The operating conditions employed for glassy and rubbery membranes are different since the main mechanism of transport is different. The former is governed by diffusivity, whereas the latter involves a solubility mechanism. Furthermore, the rubbery polymers is lacking in terms of selectivity and have lower overall performance [28].

## 2. Theory

The importance of operating temperature determination for the selected membrane material for gas separation application is related to its transport properties which are diffusion and solubility. One of the main vital phenomena that happens in gas separation membranes is the Joule Thompson effect. Theoretically, a gas flows through a membrane from the high-pressure side (subscript 1) to the low-pressure side (subscript 2), and it is assumed to happen adiabatically (no heat transfer (q = 0) and the whole system is isolated) and isenthalpic (constant enthalpy). Hence, the internal energy (*U*) change of this process is:

(1) $$\Delta U=U_{2}-U_{1}=-P_{2\;}V_{2}+P_{1\;}V_{1}$$

(2) $$U_{1}+P_{1\;}V_{1}=U_{2}+P_{2\;}V_{2}$$

(3) $$H_{1}=H_{2}$$

The temperature change in this process is called JT coefficient ($\mu_{JT}$) and is represented by the differential equation ∂T∂PH. The $\mu_{JT}$ of CO_2_ gas is 1.11 K/bar, while for CH_4_ and N_2_ it is 0.70 and 0.25 K/bar, respectively. This demonstrates that when CO_2_ is removed at high pressure, it causes a significant temperature decrease. Researchers must monitor the temperature changes caused by CO_2_ removal to ensure that the membrane performance is maintained. It is also crucial to ensure the membrane material of choice can sustain the possibility of liquid formation resulting from the temperature drop to the dew point of the feed gas. Therefore, studying membrane performance with respect to temperature variation is imperative.

The operating temperature and the gas diffusion coefficient relationship in polymeric membranes can be illustrated by the Arrhenius equation [18]:

(4) $$D_{A}=D_{AO}EXP\lfloor \frac{-E_{D}}{RT}\rfloor$$

where *D_A_* denotes the diffusion coefficient (cm^2^/s) of gas A. *D_A0_*, *E_D_*, *T*, and *R* are the pre-exponential factor, the activation energy of diffusion (in kJ/mol), the temperature (in K), and the universal gas constant, respectively. Here, the value of the *R* is 8.31 J/mol. 

Equally, the Van’t Hoff equation describes solubility as a function of temperature [29].

(5) $$S_{A}=S_{AO}EXP\lfloor \frac{-\Delta H_{S}}{RT}\rfloor$$

*S_A0_* and Δ*H_S_* are the pre-exponential factors and the enthalpy of the solution of gas *A*, respectively. The gas permeability can be stated as a function of temperature as follows:

(6) $$P_{A}=P_{AO}EXP\rangle \frac{-E_{P}}{RT}\rfloor \;E_{P}=E_{D}+\Delta H_{S}$$

*P_A0_* and *E_P_* are the pre-exponential factor, and the activation energy of permeation, respectively.

It is worth noting that as temperature rises, so do permeation and diffusion parameters, but the solubility relationship reverses [30].

## 3. Experimental

### 3.1. Materials

The hollow fiber membranes were prepared from a dope solution composed of PSf from Solvay Advanced Polymers, and a mixture of solvent and alcohol bought from Sigma-Aldrich (St. Louis, MO, USA) with appropriate composition. The name and composition of the chemicals used in this study are not mentioned in accordance with the company’s unpublished confidential information guideline. The polymer pellets were dried at 60 °C in a vacuum oven overnight before use, while solvents and non-solvent were used as received. 

### 3.2. Membrane Characterization

FESEM (S4800, Hitachi, Japan) is used to study the resultant membrane morphology for the cross-sectional image. The sample is sputter-coated with an ultrathin conductive layer, namely titanium, at high vacuum evaporation to ensure the sample is electrically conductive. This pre-treatment could avoid the accretion of a static electric field at the sample induced by electron irradiation employed during the measurement process. The membrane samples are cut into square shape sheets of size 0.5 cm × 0.5 cm and attached to a sample stub using carbon tape. For cross-sectional morphology, the membranes are fractured in liquid nitrogen and placed on a sample stub using carbon tape. Scanning is conducted at a magnification between ×500 and ×10 k.

### 3.3. Membrane Performance Testing

**Figure 1:**
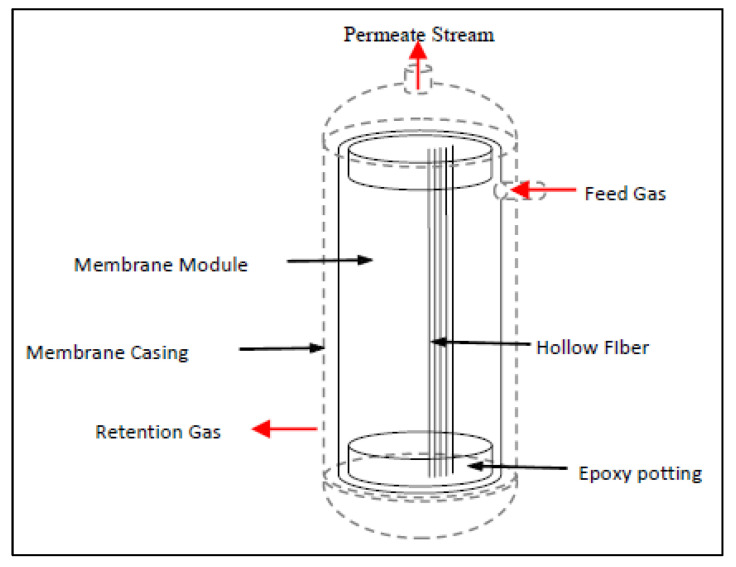
Membrane module used in this experiment.

The hollow fiber membrane module made from stainless steel (SS 316) is used in this study (refer to Figure 1). The membrane element is installed in the membrane casing that comprises of two removable parts. Rubber O-rings are used to ensure pressure-tight sealing between the tube sheet and the casing at both ends.

The prepared hollow fibers are put in the bundle and potted into Swagelok stainless steel tubing. The leak test is performed to ensure the integrity of the fiber and module prepared. Pure gas testing is conducted at low pressure (3 to 7 barg) using the bubble test method to determine the membrane ideal and intrinsic CO_2_ permeance and selectivity. Mixed gas testing is later conducted with variations of feed pressure (20 to 50 barg), temperature variation (25 to 45 °C), and CO_2_ percentage variation (15%, 20%, 40% and 70%) to determine the membrane performance close to real field environment conditions. Both pure and mixed gas measurements are determined using a permeation apparatus that allows the measurement of permeability and selectivity at various operating conditions. For the mixed gas testing, the feed, non-permeate and permeate stream gaseous composition are analyzed using gas chromatography. In this study, Micro GC Agilent 490 (Agilent Technologies, Santa Clara, CA, USA) is used for the gas composition measurement. Mass flow controller is also needed for the mass balance calculation.

CO_2_ permeance is calculated by using the following equations.

(7) $$\Delta P_{x}=\Delta P_{FR_{x}\;}-P_{P_{x}}$$

(8) $$\Delta P_{FR_{x}\;}=\frac{P_{F\;}X_{F_{x\;}}-P_{R\;}X_{R_{x\;}}\;}{In\left(\frac{P_{F\;}X_{F_{x\;}}}{P_{R\;}X_{R_{x\;}}}\right)}$$

(9) $$P_{Px}=P_{P\;}X_{P_{x}}$$

Selectivity is measured using the below equation.

(10) $$\propto \left(\frac{x}{y}\right)=\frac{{\left(\frac{P}{l}\right)}_{x}}{{\left(\frac{P}{l}\right)}_{y}}$$

where:

${\left(\frac{P}{l}\right)}_{x}$ = Permeance of x component (cm^3^/s·cmHg·cm^2^)
$Q_{x}$ = Permeate flowrate of x component (cm^3^/s)
$P_{x}$ = Differential pressure of x component (cm Hg)
A= Membrane active area (cm^2^)
*x*, *y* = Component
$P_{x}$ = Differential pressure of x component (barg)
$P_{FR_{x}\;}$ = Mean differential difference of component x between feed and retentate (barg)
$P_{F\;}$ = Feed pressure (barg)
$X_{F_{x\;}}$ = Component x concentration in feed stream (mol%)
$P_{R\;}$ = Retentate pressure (barg)
$X_{R_{x\;}}$ = Component x concentration in retentate stream (mol%)
$P_{Px=}$ Permeate component x pressure (barg)
$P_{P}$ = Permeate pressure (barg)
$X_{P_{x}}$ = Component x concentration in permeate stream (mol%).

The operating parameters are selected based on the real application needs. Operating pressure of 40 barg is usually applied in commercial field applications, and 20 barg has also been tested in this study as the lowest possible operating pressure, although it is not quite practical for the natural gas sweetening process; 50 bar is provided to provide a comparison with 40 barg data, and 30 barg data can be interpolated from 20 barg and 40 barg data.

This study focuses on mixed gas testing because the transport behavior of one component through the membrane is affected by the presence of other penetrants, which can be termed as coupling effect or competitive sorption. Permeation properties of multi-component gas mixtures are still lacking in the literature due to the complexity of conducting the testing, difficulty in controlling the operating parameters, and simultaneous analysis of multi-component [14]. Computational molecular modeling could reduce the amount of tedious and complex testing that needs to be conducted since a thorough screening can be first performed through simulation [26].

Mixed gas compositions used in this study are listed in Table 1:

For the long-term performance study, the membrane is subjected to 40% mixed gas composition at 40 barg operating pressure and 45 °C operating temperature using a system that can recycle the feed gas.

Relative numbers are the value which is relative to the reference value. The relative change is used in this paper basically to compare the trends. This comparison is generally expressed as the ratio and this number does not have any units. The actual membrane performance in this study is not mentioned in accordance with the company’s unpublished confidential information guideline.

## 4. Results and Discussion

### 4.1. Membrane Characterizations

**Figure 2:**
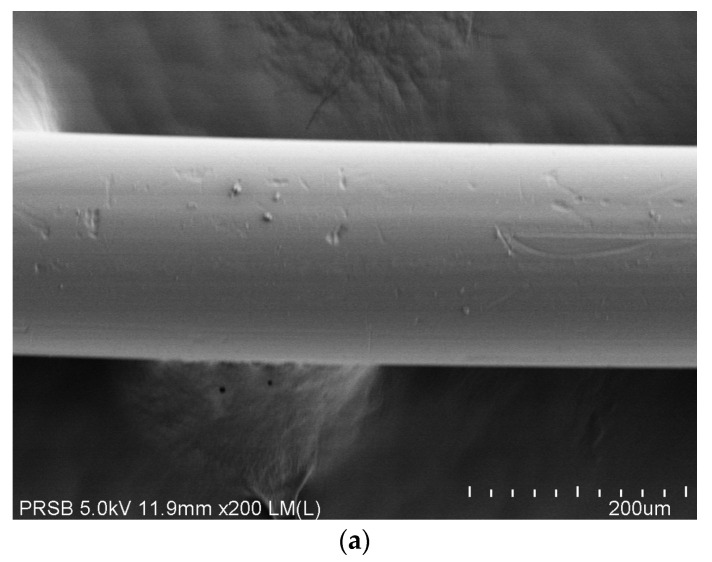

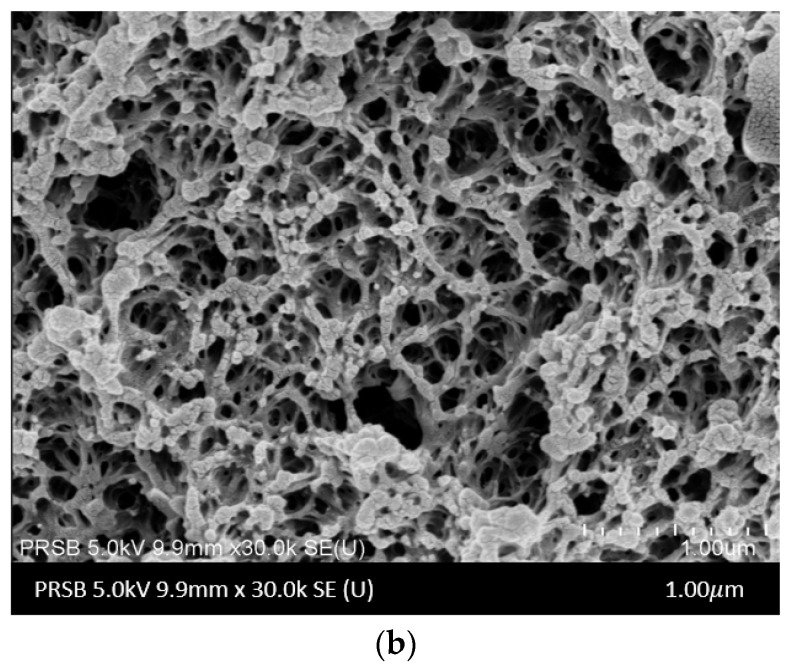
Surface (**a**) and cross-sectional (**b**) morphology FESEM image of the hollow fiber membrane fabricated in this study.

The integrally skinned asymmetric membrane with a spongy structure and thin dense skin layer is observed in membrane cross-section images, as shown in Figure 2. The desired membrane structure for gas separation has successfully been formulated and fabricated for further performance evaluation, which will be explained in the next sections. No finger-like structure or macro void formation can be found, which is detrimental for high-pressure applications. Macro void, a large pore, can frequently be detected in asymmetric membranes. However, this structure is only applicable for liquid separation, such as ultrafiltration, and it is also very useful in drug delivery systems, but for reverse osmosis process and gas separation, this macro void structure is not favorable [12].

The membrane performances in terms of CO_2_ permeance and CO_2_/CH_4_ selectivity have been studied based on several important parameters, as discussed in the following sections.

### 4.2. Effect of the Feed Pressure on the CO_2_ Permeation of the PSf Hollow Fibre Membranes at Fixed Operating Temperature

Feed pressure provides the key driving force for the gas separation membrane [2]. The determination of feed pressure is important for the equipment pipe rating, the pre-treatment and post-treatment requirements, as well as the cost of booster and export compressors. Techno-economic evaluation needs to be performed before selecting the optimum operating pressure for the process design. Type 2 polymer exhibit decreasing permeability with increasing pressure, and the permeability of gas begins to increase as the pressure reaches the CO_2_ plasticization pressure [31]. Too high operating pressure will result in a supercritical gas condition that should also be avoided. In this condition, CO_2_ exhibits properties and behaviors between liquid and gas, where the density is liquid-like while the diffusivity, surface tension, and viscosity are gas-like. Supercritical CO_2_ is considered an organic solvent with remarkable solvation power, which should be avoided when used with polymeric membranes.

**Figure 3:**
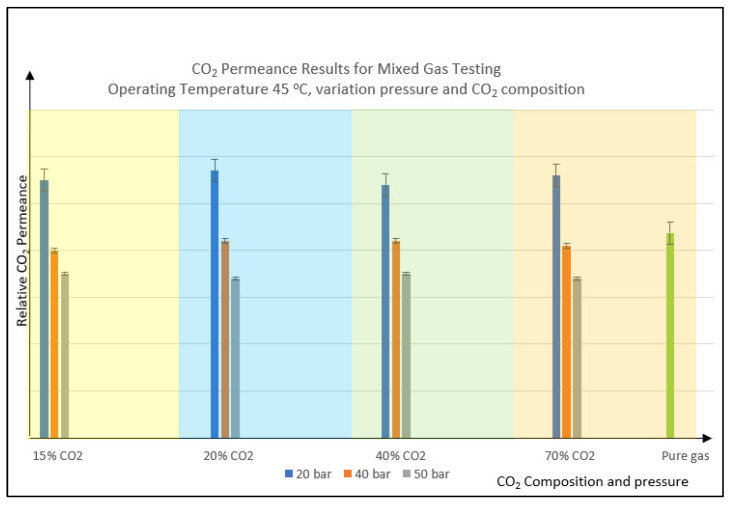
CO_2_ permeance result for mixed gas testing vs. variation of CO_2_ composition and feed pressure. Note: Pure gas testing condition is 25 °C with 3 to 7 barg applied pressure.

In this experimental analysis, the impact of CO_2_ permeance with respect to the CO_2_ composition and feed pressure changes has been investigated at a fixed operating temperature of 45 °C. As the driving force is increased from 20 to 50 barg, it can be seen in Figure 3 that the CO_2_ permeance reduced significantly for all the trends despite the variation in CO_2_%. This can be explained by several phenomena that took place in the membrane during the separation process, namely competitive sorption, pore blocking, site saturation, aging, and compaction, which greatly affect the membrane performance. These phenomena have overcome the higher-pressure driving force that is supposed to enhance the permeation. There are many mechanisms leading to free volume loss, and numerous theories have been invented to define the aging process in terms of free volume reduction [12]. This has negatively impacted the membrane and explains the declining trends shown in the graph, since the transport behavior of PSf is diffusivity dominant. In this case, if CO_2_ plasticization is more dominant than aging and compaction, the CO_2_ permeation trend should increase as the pressure increases, as is typical for cellulose-based membranes. This is because plasticization will swell the membrane, while aging and compaction will make the membrane denser. These two effects counterbalance each other, but for the cellulose case, which has lower plasticization pressure, the plasticization effect is greater than aging and compaction, as can be seen in their increasing permeability trends during the testing or operation.

However, in this case, since PSf has high plasticization pressure, aging and compaction are more dominant, leading to the decreasing trend of CO_2_ permeation. The permeability coefficient of gases at higher feed pressure usually does not obey the dual mode sorption model due to plasticization phenomena happening in the polymer [32].

The trend for CO_2_ permeance with pressure increment is subject to the polymer material. Ismail and Lorna [31] explained the three types of permeability trends for several types of glassy polymers. Type 1 is for polymers such as PSf and polycarbonate, which demonstrate a reducing permeability trend throughout the testing pressure up to 30 barg. Type 2 polymers exhibit decreasing permeability with increasing pressure, and the permeability of gas begins to increase as the pressure reaches the CO_2_ plasticization pressure. An example of a type 2 polymer is polyimide which follows dual mode sorption behavior. Type 3 polymers such as cellulose acetate and polystyrene experience an increasing permeability trend from the beginning of the testing as they have low CO_2_ plasticization pressure and behave as rubbery permeation behavior with low selectivity [33]. At the same CO_2_ percentage, increasing the pressure from 20 to 50 barg, increased the CO_2_ partial pressure and the CO_2_ solubility of the membrane material. Furthermore, the CO_2_ plasticization effect, dilates the membrane, which also counterbalances the shrinkage effect from aging, compaction, and site saturation. This, in turn, increases the permeability when the dilation is more than the shrinkage. In this case, it will be advantageous for Type 2 and Type 3 membrane materials if the selectivity is still within an acceptable level and does not drop significantly. The increasing trend of CO_2_ permeation is helped by the solubility coefficient that is increased with the increment of pressure. In this condition, membrane densification happens and hinders diffusion, but solubility can still happen through the polymer matrix. As for the PSf, it has higher CO_2_ plasticization pressure, thus, it follows the Type 1 trend when the pressure is increased, which explains the trend in Figure 3.

### 4.3. Effect of the Feed Pressure on the CO_2_/CH_4_ Selectivity of the PSf Hollow Fiber Membranes at Fixed Operating Temperature

**Figure 4:**
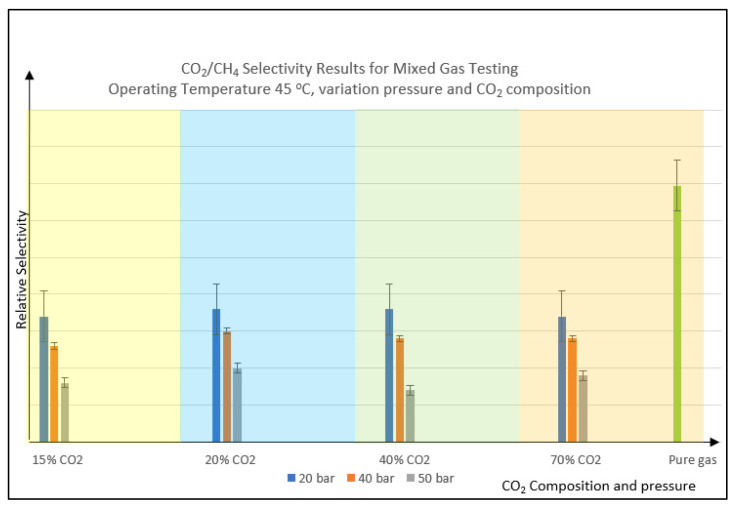
CO_2_/CH_4_ selectivity result for mixed gas testing vs. variation of CO_2_ composition and feed pressure. Note: Pure gas testing condition is 25 °C temperature with 3 to 7 bar applied pressure.

The same trend for selectivity is observed when the pressure is increased from 20 to 50 barg, where the trends show a decreasing trend for all CO_2_% variation, as displayed in Figure 4. The selectivity values for all the mixed gas testing are lower than pure gas values. This is because the pressure force also pushes the slower gas i.e., methane to permeate. The increasing pressure has resulted in both reductions of CO_2_ permeance due to compaction and aging and reduction of selectivity for PSf membrane that has diffusivity as the transport dominant factor.

### 4.4. Effect of the Feed Temperature on the CO_2_ Permeation of the PSF Hollow Fiber Membranes at Fixed Operating Pressure

Feed temperature is one of the most significant parameters for membrane operation as it affects the transport properties. The impact is more prominent for diffusivity-dominan polymers, where diffusion increases with rising temperature, whereas for solubility-dominating membranes, the effect is reversed, with increasing temperature lowering solubility. As mentioned by Duthie diffusion at high temperatures is mostly contributed by Henry’s law species and plasticization effects are conquered by Henry’s law dissolution [6]. At lower temperatures, the movement of Langmuir component species also contributes to the total diffusion coefficient [6].

Operating temperature also considerably impacts the robustness of the membrane if the setting is below the feed gas dewpoint after the CO_2_ removal and liquid are allowed to form during operation. The liquid is formed when condensation happens in the gas separation membrane during the separation of CO_2_ from natural gas. There are two effects that will result in condensation. Firstly, the bulk CO_2_ removal that permeates faster than the heavy hydrocarbon makes the gas heavier, and the dew point increases towards the operating temperature. Secondly, the gas cools down due to Joule Thompson (JT) effect as it passes through the membrane. The change in fluid temperature happens upon expansion (pressure decrease), causing a significant change to the temperature between the feed gas, retentate gas, and the permeate gas. The drastic temperature change results in liquid formation and greatly affects the permeation properties in which the permeance is reduced by pore blocking. On the other hand, the selectivity is enhanced due to the hydrocarbon permeation is also blocked by the presence of liquid. In many cases, polymers are contacted with liquids that penetrate their structure and plasticize it [7], and in this case, the selectivity will drop.

Experimental studies have been performed to explore the impact of operating temperature changes on the formulated membrane performance. By doing this investigation, the optimum temperature can be set to obtain the best performance while avoiding liquid formation by providing sufficient superheating. In this experiment, condensation cannot be simulated, and it requires heavier gases in the feed gas to demonstrate the liquid formation usually found in the field.

**Figure 5:**
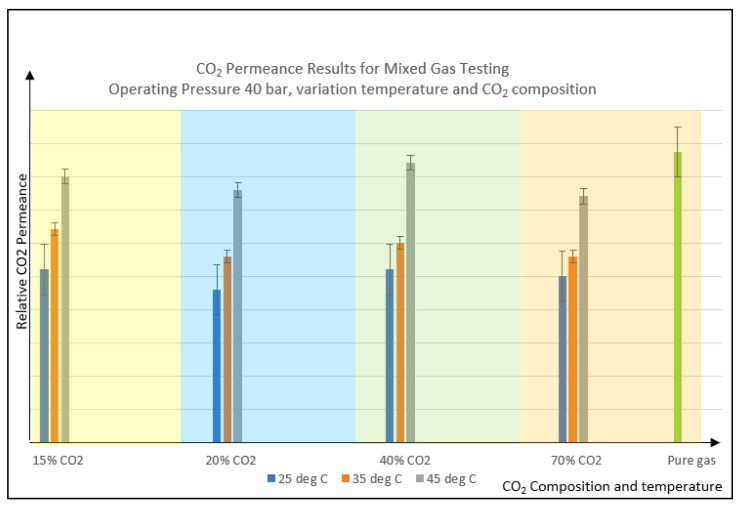
CO_2_ permeance result for mixed gas testing vs. variation of CO_2_ composition and feed temperature. Note: Pure gas testing condition is 25 °C temperature with 3 to 7 barg applied pressure.

Based on Figure 5, when the temperature increases, the increasing trend can be seen for the CO_2_ permeance for all cases of CO_2_ percentage following the Arrhenius equation [2]. However, the mixed gas number is lower than the pure gas value. Similar findings have been reported by other researchers [2,31,34]. This is because, at higher temperatures, the diffusion increases as the gas molecule’s kinetic energy and gas collision increases beyond its minimum activation energy, especially for the fast gas. This is advantageous for the polymers governed by diffusivity as the dominant transport behavior. Conversely, the solubility has an opposing trend with the temperature, where the higher the temperature, the lower the solubility. When the increase in diffusivity is higher than the decrease in solubility for a given polymer material, the overall permeability is higher.

At high temperatures, the polymer matrix is also hypothesized to increase segmental mobility resulting in easier penetration of the fast gas through the thin skin layer of an asymmetric membrane. This shows that a higher temperature can be set as this membrane operating temperature to gain the benefit of higher CO_2_ permeance with a certain limit to control the other effect, such as aging. Further investigation is needed for the long-term effect under continuous exposure of the polymer to higher pressure and temperature. At higher operating temperatures, perm selectivity is shifted to a higher permeability value [35]. The best gas permselective membrane is reached when high selectivity combined with high permeability can be attained simultaneously [14].

**Figure 6:**
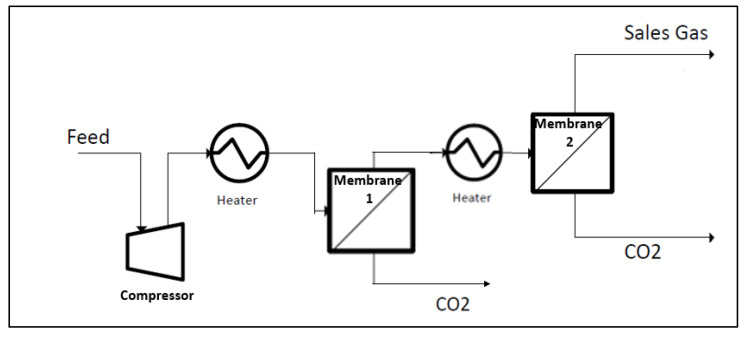
Illustration showing the hot process system for membrane operation to avoid liquid condensation.

Condensation can be prevented for membrane materials that are sensitive to liquid hydrocarbon by heating the feed gas and inter-step membrane (refer to Figure 6) to the optimum operating temperature. This is to provide a sufficient margin of superheat based on the predetermined dew point value that can prevent the membrane from being affected by the liquid formation. Therefore, a higher operating temperature operation is favorable for the membrane that is not robust to liquid and has diffusivity as the dominant transport mechanism. However, the impact of aging and other potential issues due to long-term operation at high temperatures needs to be further investigated.

### 4.5. Effect of the Feed Temperature on the CO_2_/CH_4_ Selectivity of the PSf Hollow Fiber Membranes at Fixed Operating Pressure

**Figure 7:**
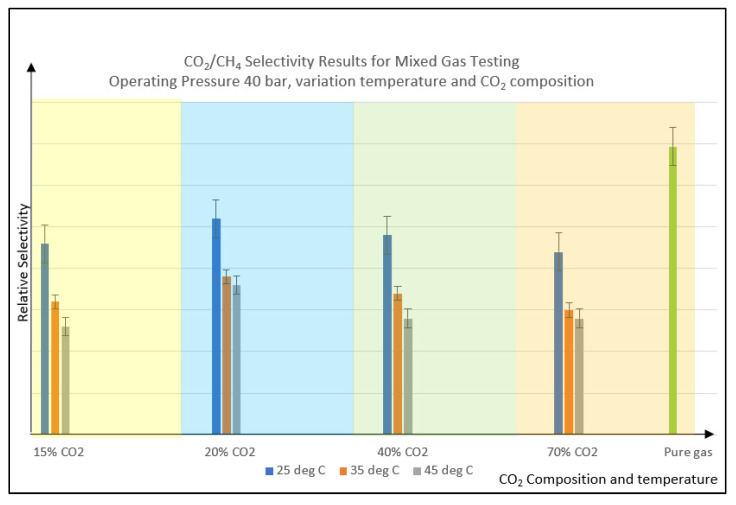
CO_2_/CH_4_ selectivity result for mixed gas testing vs. variation of CO_2_ composition and feed temperature. Note: Pure gas testing condition is 25 °C temperature with 3 to 7 barg applied pressure.

When the temperature is increased from 25 to 45 °C, the selectivity decreases for all the CO_2_% variations, as shown in Figure 7. The decrease in selectivity can be explained by the kinetic energy and diffusivity increment for both fast and slow gas, i.e., CO_2_ and CH_4,_ as the temperature is increased. The selectivity is reduced because the CH_4_ gas also has higher activation energy at higher temperatures, which increases permeation. The competition impact between CH_4_ and CO_2_ has resulted in a declining trend in selectivity value as the temperature increases [2]. This is why membranes with high selectivity at room temperature may produce low separation at elevated temperatures [35]. Low-temperature operation could minimize the amount of ‘slow gases’ permeation through the membrane since they have lower energy. There is a tradeoff between CO_2_ permeability and selectivity that needs to be considered when applying high-temperature application. If the selectivity value at high-temperature operation is still at an acceptable limit, then the setting can be used for the continuous membrane operation application.

### 4.6. Effect of the Feed Composition on the CO_2_ Permeation and Selectivity of the PSf Hollow Fiber Membranes

**Figure 8:**
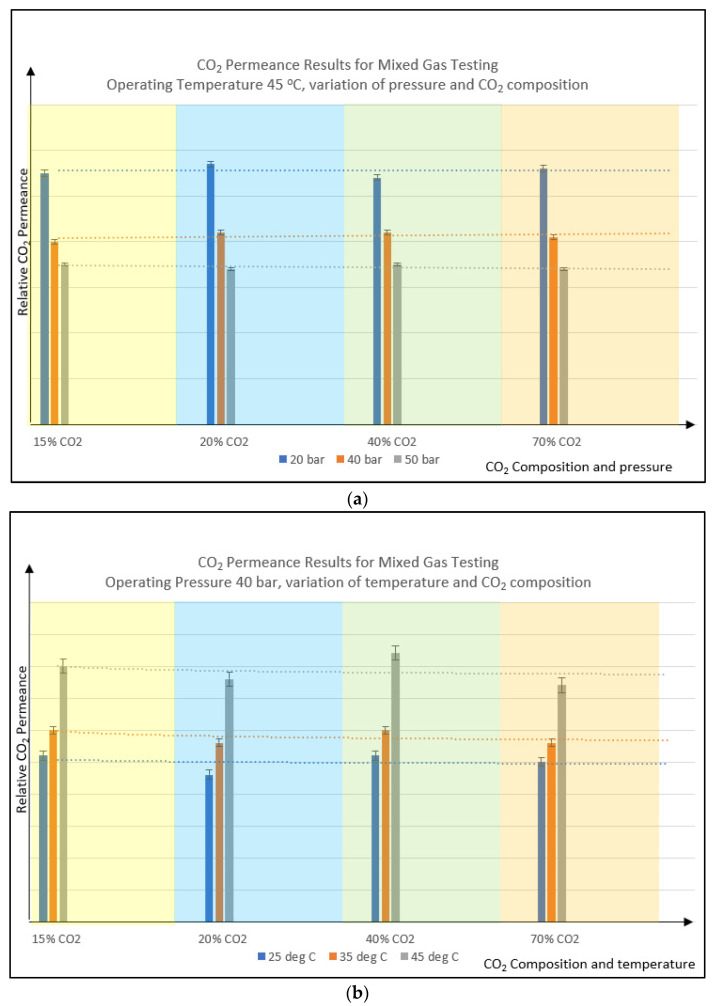
Relative CO_2_ permeance vs. variation of CO_2_ feed composition; varies operating pressure (**a**) and varies operating temperature (**b**). Dotted line is the trendline as a guide.

Based on Figure 8a,b, it could be summarized that the CO_2_ permeance trend is quite stable for the PSf membrane formulated in this study. This happens even though the CO_2_ partial pressure is increased with the increment of CO_2_ composition [26] from 15% to 70%, within the same operating pressure of 40 barg. Higher CO_2_ concentration at the same operating pressure leads to higher CO_2_ partial pressure, which is supposed to boost CO_2_ permeance to a certain extent. Plasticization is expected to happen following the CO_2_ concentration level as CO_2_ is a strong plasticizer agent. Using a polyimide membrane, Duthie found that as the concentration of CO_2_ is boosted to a higher value, the polymer swells due to polymer relaxation, leading to an increase in the diffusion coefficient [6]. However, for the PSf membrane prepared in this study, demonstrated higher polymer rigidity and strong plasticization resistance to CO_2_ gas, as well as a stable trend as the CO_2_% increased.

According to Fick’s Law, diffusion increases as the concentration gradient increases, and as per Henry’s law, the solubility increases as the partial pressure increases. It is noted that at higher CO_2_ composition, the effect of plasticization will become more severe. However, since the material used in this study is PSf which is known to have very high plasticization pressure [36], the highest condition used in this experiment, which is 40 barg with 70% CO_2_, resulting in 28 barg of CO_2_ partial pressure. This partial pressure is still below the plasticization pressure of PSf, which is around 34 barg, making the trend looks stable for both permeability and selectivity as the CO_2_ increased from 15% to 70%. Under this condition, most membrane materials would have experienced certain degrees of plasticization.

**Figure 9:**
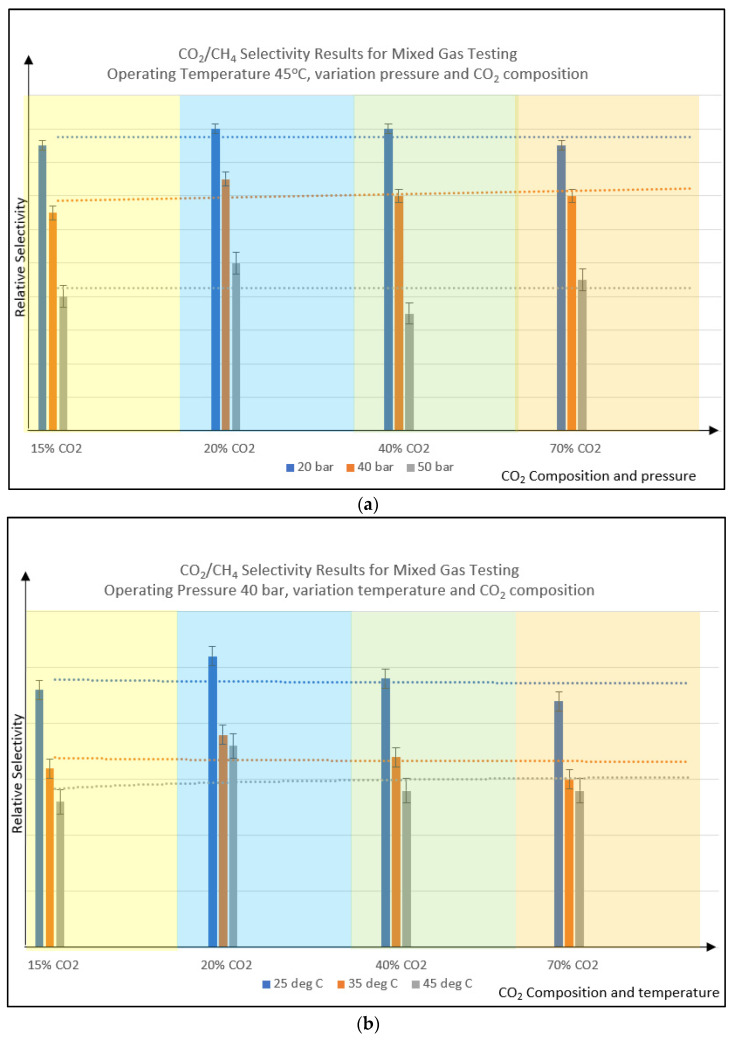
Relative selectivity vs. variation of CO_2_ feed composition; varies operating pressure (**a**) and varies operating temperature (**b**). Dotted line is the trendline as a guide.

Based on Figure 9a,b, it could be summarized that the selectivity trend is quite stable for both variations of pressure (constant temperature) and variation of temperature (constant pressure) for the formulated PSf membrane used in this study. This happened even though the CO_2_ partial pressure is increased with the increment of CO_2_ composition [26] from 15% to 70%, within the same operating temperature of 45 °C. This shows the integrity and rigidity of the PSf membrane as the pressure increased, reflecting its higher plasticization pressure.

### 4.7. Long-Term Performance Study of the Membrane

A long-term performance study is performed to investigate the stability of the membrane performance over time. It has been well-established that thin films aged faster than thick films [26]. As plasticization is also a time-dependent phenomenon, the degree of plasticization needs to be further investigated, if any. Normally, physical aging takes place slowly over a long period of time, with the membrane gradually approaching equilibrium from the initial non-equilibrium state induced during the fabrication stage [12].

**Figure 10:**
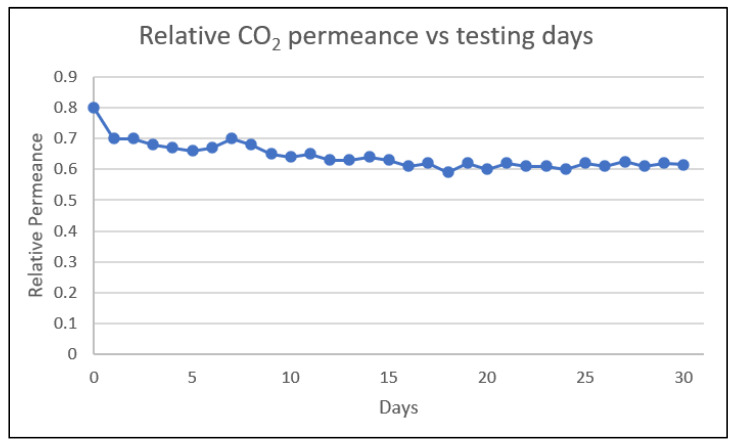
Relative CO_2_ permeance vs. testing days trend showing the membrane stability.

Based on Figure 10, throughout 30 days of testing with 40% mixed gas at 40 barg and 45 °C, the membrane performance is stable with only a minor decline due to compaction and aging. The graph achieved a plateau trend on the 15th day, indicating the high stability of the membrane. White mentioned that the membrane films from multiple polymers, for example, PSf, polyimide Matrimid, and PPO (polyphenylene oxide) with 0.4 to 60 microns in thickness, were periodically tested in a laboratory for up to 10,000 h [26]. He had shown a substantial reduction in permeability trend when those membranes were stored at ambient conditions while not in testing instead of keeping them at constant conditions [26]. Ma and Koros proposed that the frequent changes in CO_2_ partial pressure for the asymmetric hollow-fiber membranes affect the long-term performance of the membrane as compared to steady and constant CO_2_ exposure which will yield more stable membrane performance [37]. This can be further investigated in field conditions, which will have some fluctuations due to operational issues and sudden trips or unplanned shutdowns. Constant condition is proposed to ensure long-term membrane performance; hence proper procedure needs to be in place if the fluctuation happens.

**Figure 11:**
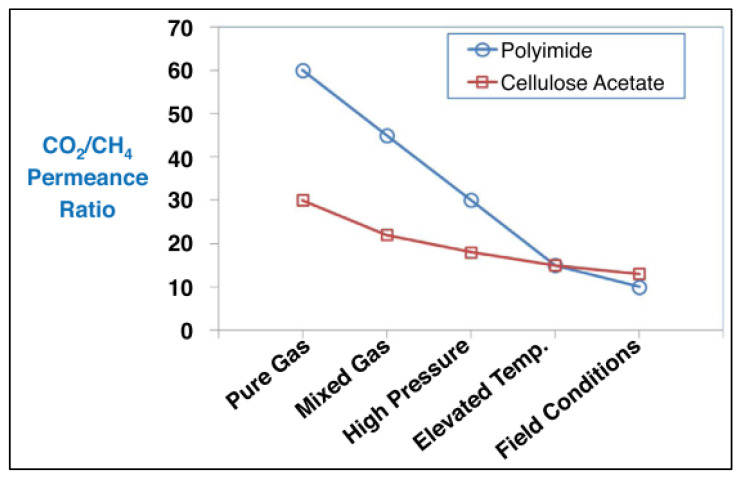
Illustration of PI (Polyimide) and CA (Cellulpse Acetate) membrane trend going from pure gas testing to the field conditions testing results [26].

As shown in Figure 11, some polymers with high separation performance in laboratory testing degrade more drastically than conventional polymers such as polyimide and cellulose acetate membrane [26,38]. This can be explained by the presence of contaminants in natural gas. This effect must be considered when one starts to formulate a new membrane. The contaminants in gas fields are H_2_S, heavy hydrocarbon [39], aromatic, mercury, water, and many others. Investigation using mixed gas with 5 components is still not enough and further study is needed with cyclic testing to test the membrane durability, robustness, and lifetime, which is critically important in maintaining the high performance of the membrane formulated in the laboratories.

Heavy hydrocarbon contamination, even at low concentrations, is hypothesized to be the reason for some membrane failures and loss in performance. According to the dual mode sorption theory, highly condensable feed components, such as toluene, will contribute to severe competition impact due to their size and based on its critical temperature, it can be easily condensed at the operating pressure applied. Toluene can block permeation opportunities of CO_2_ and made the membrane performance drop [39,40,41,42,43,44,45,46]. The impact of heavy hydrocarbon, including aromatic hydrocarbon, on the membrane system, has been lacking due to the complexity of the components present and the difficulty in characterizing their effects experimentally [39]. Heavy hydrocarbon, including aromatic hydrocarbon, also can severely swell and plasticize the polymer.

## 5. Conclusions

Commonly, the permeation behavior of gases through membranes is related mainly to the properties of gases and membranes along with the operating conditions [14]. Based on the study results, it can be concluded that the formulated PSf membrane has a decrease in CO_2_ permeation as pressure rises due to gas diffusivity being blocked. Increasing the operating temperature can help to boost CO_2_ diffusion and permeation. High productivity can be attained at higher operating temperatures with a reduction in product purity. At higher partial pressure conditions, when the CO_2_ percentage is increased, the CO_2_ productivity and product purity remain stable due to the rigidity and integrity of the membrane produced. Interestingly, since PSf has higher plasticization pressure, it is unaffected by changes in CO_2_ percentage up to 70% CO_2_. The present study shows that the membrane material formulated has the potential for further evaluation at the field stage. Longer testing duration at the optimum operating conditions on site is needed with the real feed gas, which includes heavy hydrocarbon and aromatic components, to confirm the actual membrane lifetime, robustness, durability, reliability, and stability.

## Figures and Tables

**Table 1:** Gas composition used in this study.

Gas/Composition (%)	CO_2_	C_1_	C_2_	C_3_	N_2_
Set 1	15.00	76.88	3.42	2.65	2.05
Set 2	20.00	71.88	3.42	2.65	2.05
Set 3	40.00	55.90	2.00	1.60	0.50
Set 4	70.00	28.40	1.00	0.40	0.20

## References

[1] Mordor Intelligence Gas Separation Membrane Market: Growth, Trends, Covid-19 Impact and Forecasts (2022–2027). https://www.mordorintelligence.com/industry-reports/gas-separation-membrane-market.

[2] Hwang H.Y., Nam S.Y., Koh H.C., Ha S.Y., Barbieri G., Drioli E. (2012). The effect of operating conditions on the performance of hollow fiber membrane modules for CO_2_/M_2_ separation. J. Ind. Eng. Chem..

[3] Jia B., Chen Z., Xian C. (2022). Investigations of CO_2_ storage capacity and flow behavior in shale formation. J. Pet. Sci. Eng..

[4] Hawthorne S.B., Grabanski C.B., Jin L., Bosshart N.W., Miller D.J. (2021). Comparison of CO_2_ and Produced Gas Hydrocarbons to Recover Crude Oil from Williston Basin Shale and Mudrock Cores at 10.3, 17.2, and 34.5 MPa and 110 °C. Energy Fuels.

[5] Kazemi M., Borujeni T. Molecular Dynamic Study of Carbon Dioxide in Carbon Based Organic Nanopores.pdf. Proceedings of the Society of Petroleum Engineers—Abu Dhabi International Petroleum Exhibition and Conference.

[6] Carta M., Bernardo P. (2015). Gas Separation by Membrane Operations. Encyclopedia of Membranes.

[7] Duthie X., Kentish S., Powell C., Nagai K., Qiao G., Stevens G. (2007). Operating temperature effects on the plasticization of polyimide gas separation membranes. J. Membr. Sci..

[8] Wypych G. (2017). Handbook of Plasticizer.

[9] Li P., Hosseini S.S., Zhang M., Deng L., Xiang D., Cao B. (2019). Approaches to Suppress CO_2_-Induced Plasticization of Polyimide Membranes in Gas. Processes.

[10] Bos A., Pünt I.G.M., Wessling M., Strathmann H. (1999). CO_2_-induced plasticization phenomena in glassy polymers. J. Membr. Sci..

[11] Liu Y., Liu Z., Morisato A., Bhuwania N., Chinn D., Koros J. (2020). Natural gas sweetening using a cellulose triacetate hollow fiber membrane illustrating controlled plasticization benefits. J. Membr. Sci..

[12] Alaslai N., Ghanem B., Alghunaimi F., Litwiller E., Pinnau I. (2016). Pure- and mixed-gas permeation properties of highly selective and plasticization resistant hydroxyl-diamine-based 6FDA polyimides for CO_2_/CH_4_ separation. J. Membr. Sci..

[13] Houben H.J.M., Borneman Z., Nijmeijer K. (2020). Plasticization behavior of crown-ether containing polyimide membranes for the separation of CO_2_. Sep. Purif. Technol..

[14] Minelli M., Oradei S., Fiorini M., Sarti G.C. (2019). CO_2_ plasticization effect on glassy polymeric membranes. Polymer.

[15] Yong W.F., Kwek K.H.A., Liao K.S., Chung T.S. (2015). Suppression of aging and plasticization in highly permeable polymers. Polymer.

[16] Huang Y., Wang X., Paul D.R. (2006). Physical aging of thin glassy polymer films: Free volume interpretation. J. Membr. Sci..

[17] Genduso G., Pinnau I. (2020). Quantification of sorption, diffusion, and plasticization properties of cellulose triacetate films under mixed-gas CO_2_/CH_4_ environment. J. Membr. Sci..

[18] Sadrzadeh M., Shahidi K., Mohammadi T. (2009). Effect of operating parameters on pure and mixed gas permeation properties of a synthesized composite PDMS/PA membrane. J. Membr. Sci..

[19] Zhang C., Yan J., Tian Z., Liu X., Cao B., Li P. (2017). Molecular Design of Troger’s Base-Based Polymers Containing Spirobichroman Structure for Gas Separation. Ind. Eng. Chem. Res..

[20] Saimani S., Dal-cin M.M., Kumar A., Kingston D.M. (2010). Separation performance of asymmetric membranes based on PEGDa/PEI semi-interpenetrating polymer network in pure and binary gas mixtures of CO_2_, N_2_ and CH_4_. J. Membr. Sci..

[21] Achoundong C.S.K., Bhuwania N., Burgess S.K., Karvan O., Johnson J.R., Koros W.J. (2013). Silane Modification of Cellulose Acetate Dense Films as Materials for Acid Gas Removal. Macromolecules.

[22] Adewole J.K., Sultan A.S. (2019). Polymeric Membranes for Natural Gas Processing: Polymer Synthesis and Membrane Gas Transport Properties. Functional Polymers, Polymers and Polymeric Composites.

[23] Favvas E.P., Katsaros F.K., Papageorgiou S.K., Sapalidis A.A., Mitropoulos A.C. (2017). A review of the latest development of polyimide based membranes for CO_2_ separations. React. Funct. Polym..

[24] Farahdila K., Goh P.S., Ismail A.F., Wan N.F.W.M., Mohd H.M.H., Soh W.K., Yeo S.Y. (2021). Challenges in Membrane Process for Gas Separation from Natural Gas. J. Appl. Membr. Sci. Technol..

[25] Visser T., Masetto N., Wessling M. (2007). Materials dependence of mixed gas plasticization behavior in asymmetric membranes. J. Membr. Sci..

[26] Falbo F., Tasselli F., Brunetti A., Drioli E., Barbieri G. (2014). Polyimide hollow fiber membranes for CO_2_ separation from wet gas mixtures. Braz. J. Chem. Eng..

[27] Ricci E., Di E., Degli M., Liu L., Mensitieri G., Fabbri P., Kentish S.E., Grazia M., Angelis D. (2021). Towards a systematic determination of multicomponent gas separation with membranes: The case of CO_2_/CH_4_ in cellulose acetates. J. Membr. Sci..

[28] Miandoab E.S., Kentish S.E., Scholes C.A. (2021). Modelling competitive sorption and plasticization of glassy polymeric membranes used in biogas upgrading. J. Membr. Sci..

[29] Genduso G., Ghanem B.S., Pinnau I. (2019). Experimental mixed-gas permeability, sorption and diffusion of CO_2_-CH_4_ mixtures in 6FDA-mPDA polyimide membrane: Unveiling the effect of competitive sorption on permeability selectivity. Membranes.

[30] White L.S. (2020). Effect of operating environment on membrane performance. Curr. Opin. Chem. Eng..

[31] Natarajan P., Sasikumar B., Elakkiya S., Arthanareeswaran G., Ismail A.F., Youravong W., Yuliwati E. (2021). Pillared cloisite 15A as an enhancement filler in polysulfone mixed matrix membranes for CO_2_/N_2_ and O_2_/N_2_ gas separation. J. Nat. Gas. Sci. Eng..

[32] Dehghani Kiadehi A., Rahimpour A., Jahanshahi M., Ghoreyshi A.A. (2015). Novel carbon nano-fibers (CNF)/polysulfone (PSf) mixed matrix membranes for gas separation. J. Ind. Eng. Chem..

[33] Ilicak I., Boroglu M.S., Durmus A., Boz I. (2021). Journal of Natural Gas Science and Engineering Influence of ZIF-95 on structure and gas separation properties of polyimide-based mixed matrix membranes. J. Nat. Gas. Sci. Eng..

[34] Lokhandwala K.A., Baker R.W. (2008). Natural Gas Processing with Membranes. Ind. Eng. Chem. Res..

[35] Mushtaq A., Mukhtar H.B., Shariff A.M., Mannan H.A. (2013). A Review: Development of Polymeric Blend Membrane for Removal of CO_2_ from Natural Gas. Int. J. Eng. Technol. IJET-IJENS.

[36] Costello L.M., Koros W.J. (1995). Thermally stable polyimide isomers for membrane-based gas separations at elevated temperatures. J. Polym. Sci. Part B Polym. Phys..

[37] McKeen L.W. (2012). Introduction to Permeation of Plastics and Elastomers. Permeability Properties of Plastics and Elastomers.

[38] Adewole J.K., Ahmad A.L., Ismail S., Leo C.P., Sultan A.S. (2015). Comparative studies on the effects of casting solvent on physico- chemical and gas transport properties of dense polysulfone membrane used for CO_2_/CH_4_ separation. J. Appl. Polym. Sci..

[39] Ismail A.F., Lorna W. (2002). Penetrant-induced plasticization phenomenon in glassy polymers for gas separation membrane. Sep. Purif. Technol..

[40] Kadirkhan F., Goh P.S., Ismail A.F. (2022). Recent Advances of Polymeric Membranes in Tackling Plasticization and Aging. Membranes.

[41] Halim M.H., Kadirkhan F., Mustapa W.N.F., Soh W., Yeo S. (2020). Natural Gas Sweetening Polymeric Membrane: Established Optimum Operating Condition at 70% of CO_2_ Concentration Fedd Gas Stream. Malaysian J. Fundam. Appl. Sci..

[42] Sridhar S., Smitha B., Aminabhavi T.M. (2007). Separation of carbon dioxide from natural gas mixtures through polymeric membranes—A review. Sep. Purif. Rev..

[43] Ma C., Koros W.J. (2018). Physical aging of ester-cross-linked hollow fiber membranes for natural gas separations and mitigation thereof. J. Membr. Sci..

[44] Siagian U.W.R., Raksajati A., Himma N.F., Khoiruddin K., Wenten I.G. (2019). Membrane-based carbon capture technologies: Membrane gas separation vs. membrane contactor. J. Nat. Gas Sci. Eng..

[45] Al-juaied M., Koros W.J. (2006). Performance of natural gas membranes in the presence of heavy hydrocarbons. J. Membr. Sci..

[46] Liu Q., Galizia M., Gleason K.L., Scholes C.A., Paul D.R., Benny D. (2016). Influence of toluene on CO_2_ and CH4 gas transport properties in thermally rearranged (TR) polymers based on 3,3′-dihydroxy-4,4′-diamino-biphenyl (HAB) and 2,2′-bis-(3,4-dicarboxyphenyl) hexafluotopropane dianhydride (6FDA). J. Membr. Sci..

